# Abstract of the Proceedings of the Buffalo Medical Association

**Published:** 1863-03

**Authors:** Julius F. Miner

**Affiliations:** Secretary


ART. II.—*Abstract of the Proceedings of the Buffalo Medical Association.*

TUESDAY EVENING, February 3d, 1863.

Dr. T. T. Lockwood, President *pro tem* in the Chair. Minutes of the last meeting read and accepted.

*Dr. Lockwood* reported cases of small pox, and gave the particulars of a case where the eruption was of a peculiar character. Spoke also of the protective value of vaccination, and gave some interesting cases, in illustration of his remarks.

*Dr. Rochester* expressed his preference for vaccine lymph over the virus as contained in the scale, but said that one great obstacle in the way of its general use was the impossibility of preserving it for any great length of time. Remarked also upon the effect of heat and cold in destroying the specific qualities of vaccine virus. Had recently sent some very nice vaccine virus in the scale to Almira, where forty patients were vaccinated with it, in all of which it failed. The mail bag remaining over night in very exposed and cold place, was assigned as the cause of failure by the physician to whom it was sent.

*Dr. Ring* remarked that the scale would often appear to have lost its specific qualities, when if it was ground upon glass and thoroughly mixed, it would be found perfectly reliable, thus showing that parts of it might not contain virus, while yet other portions retained the specific characters unchanged.

*Dr. Cronyn* had noticed great differences in the results of vaccination so far as obtaining the proper effect is concerned. Vaccinated one day twelve children from ground scale; three only had fine pustules. Again eight vaccinations, with the same virus, six operated finely. Thought the condition of the patient had something to do with the failure. Dr. C. spoke also of the protective value of vaccination, and related a case where vaccination seemed to change the natural course of small pox even after the eruption was seen under the skin,

*Dr. Wyckoff* spoke of the value of vaccination, and had no doubt that it would modify small pox when its action was obtained, even though the disease was considerably advanced in its course. Remarked also upon measures for preventing pitting in small pox. Applied cloths smeared with Olive oil.

*Dr. Miner* related the following case of Hæmaturia. Mrs. C., aged 34 years, of robust and healthy appearance, had been subject, for about three years, to occasional attacks of bleeding, the blood issuing from the urethra. She was the mother of one child, about five years of age. Was regular in her menstrual secretion, both in time and quantity; did not suffer any pain either in voiding the bloody urine, or at other times. The quantity of urine was natural, as near as could be judged, and there was an entire absence of all indications of disease, except the single symptom of bloody urine. About one year since, she suffered a very severe attack, which continued for six weeks, notwithstanding every effort to arrest it, reducing the strength so greatly as to cause anxiety, and even, almost despair of her recovery. At this juncture, the hemorrhage ceased instantly: when she urinated in the morning, she was astonished to find quite healthy urine, and no blood. This sudden interruption of bleeding has been the usual manner of arrest, when she would enjoy, perhaps, some months of uninterrupted good health. Specimens of the urine had been examined by Prof. Wm. Mason, and found to consist of nearly or quite one-fourth part blood. Albumin was also present, which would be derived from the blood. Crystals of the Triple Phosphates, were present in the specimen examined, which had been standing for 48 hours previous to examination. It is, in this case, impossible to say, with any certainty, where, in the long track of mucuous membrane which might be its source, the blood arises, though the presumptive evidence will favor the conclusion that it has its origin in the kidney.

Idiopathic hæmaturia must be an exceedingly rare disease; the traumatic variety much more frequently. Cases, where the exact seat of the hæmorrhage cannot be ascertained during life, are sufficiently common; many cases are exceedingly obscure, and it is said, that even fatal hæmorrhage has occured, the blood discharged from the urethra, and yet, *post mortem* examination fail to reveal any structural change, or even show its exact origin.

This case is in many respects, a most remarkable one, and would seem to be strictly idiopathic, yet the extreme infrequency of such disease, leads to doubt, on that point. The great quantity of blood voided; the fact of no increase in frequency of urination or pain, or other inconvenience attending it, and the usual sudden manner of arrest, together with the absence of all symptoms leading to positive diagnosis, constitute the remarkable features of the case. Dr. Miner replied to inquiries from different members, that the patient was not nervous or hysterical; had no chills after voiding urine; that the blood was thoroughly mixed with the urine, and that the specific gravity of the urine was unchanged in any great degree.

*Dr. Rochester* proposed Dr. Joseph A. Peters, (now surgeon in the army,) for membership.

*Voted* on motion of Dr. Rochester, that a committee be appointed to consider the propriety of modifying the fee bill to correspond, in a measure, with the present advance in everything else, and to report at the next meeting.

Drs. Lockwood, White and Burwell, were appointed such committee.

*Voted* to adjourn to the first Tuesday evening in March.

JULIUS F. MINER, Secretary.

